# Low‐density lipoprotein receptor‐related protein 6 regulates alternative pre‐mRNA splicing

**DOI:** 10.1111/jcmm.13682

**Published:** 2018-08-01

**Authors:** Tianyou Yuan, Shiyi Wang, Chaoyue Hu, Yufei Wu, Dandan Liang, Li Li, Yi Liu, Jun Li, Yi‐Han Chen

**Affiliations:** ^1^ Institute of Medical Genetics East Hospital, Tongji University School of Medicine Shanghai China; ^2^ Heart Health Center Tongji University School of Medicine Shanghai China; ^3^ Key Laboratory of Arrhythmias of the Ministry of Education of China Tongji University School of Medicine Shanghai China; ^4^ Department of Pathology and Pathophysiology Tongji University School of Medicine Shanghai China

**Keywords:** alternative splicing, integrin, lipoprotein receptor‐related protein 6, myodifferentiation, Wnt coreceptor

## Abstract

Low‐density lipoprotein receptor‐related protein 6 (LRP6) serves as a Wnt coreceptor. Although Wnt/LRP6 signalling is best known for the β‐catenin‐dependent regulation of target genes in tissue development and homeostasis, emerging evidence demonstrates the biological aspects of LRP6 beyond a Wnt coreceptor. Whether LRP6 modulates tissue development in a Wnt/β‐catenin signalling‐independent manner remains unknown. Using a model of striated muscle development, we observed that LRP6 was almost undetectable in proliferating myoblasts, whereas its expression gradually increased in the nucleus of myodifferentiating cells. During myodifferentiation, LRP6 modulated the muscle‐specific splicing of integrin‐β1D and consequent myotube maturation independently of the β‐catenin‐dependent Wnt signalling. Furthermore, we identified that the carboxy‐terminal serine‐rich region in LRP6 bond to the adenine‐rich sequence within alternative exon D (AED) of integrin‐β1 pre‐mRNA, and therefore, elicited AED inclusion when the spliceosome was recruited to the splice site. The interaction of LRP6 with the adenine‐rich sequence was sufficient to overcome AED exclusion by a splicing repressor, polypyrimidine tract binding protein‐1. Besides the integrin‐β1, deep RNA sequencing in different types of cells revealed that the LRP6‐mediated splicing regulation was widespread. Thus, our findings implicate LRP6 as a potential regulator for alternative pre‐mRNA splicing.

## 1 INTRODUCTION

The Wnt/β‐catenin signalling is an evolutionarily conserved key regulator of tissue development and homeostasis in all animals.^1^ Its abnormality affects tissue growth and function, and can progress to a variety of diseases.^1^ Low‐density lipoprotein receptor‐related protein 6 (LRP6) has been identified as a cell‐surface Wnt coreceptor responsible for transducing the canonical β‐catenin‐dependent Wnt signalling.^2^ The Wnt/LRP6 signalling proceeds mainly through β‐catenin‐mediated transcription of target genes.^1^ Transcription‐independent Wnt/LRP6 signalling also regulates cell growth and mitotic progression, axonal remodelling and maturation of germ cells, suggesting the diversity in the action modality of Wnt/LRP6 signalling.^3^ Germline inactivation of the *Lrp6* gene in mice causes extensive organ defects and neonatal lethality, which resembles the phenotypes caused by mutations in individual *Wnt* genes.^4^, ^5^ Moreover, evidence for the pathophysiological relevance of *Lrp6* mutation and deficiency, such as Alzheimer disease, coronary artery disease and cardiac ischemia, have emerged, and presumably the aberrant Wnt signalling mediates the functional link.^2^, ^6^

The biological aspects of LRP6 beyond a Wnt coreceptor have been gradually recognized. Our recent study demonstrated a scaffolding role of LRP6 in the membrane targeting of connexin 43 and thus gap junction assembly in the mouse heart.^7^ Wan et al reported the property of LRP6 as a trafficking adaptor, showing that *Lrp6* knockdown disrupts the localization of Gαs to the plasma membrane and impairs a functional G protein‐coupled receptor (GPCR) signalling pathway for the production of cAMP.^8^ Interestingly, several lines of evidence also documented that a soluble LRP6 intracellular domain can translocate to the nucleus and directly modulates the activity of the Wnt responsive transcription factor TCF/LEF‐1,^9^, ^10^ suggesting the potential transcriptional and post‐transcriptional activities of LRP6 protein. These reports led us to suspect whether LRP6 modulates tissue development and homeostasis in a Wnt/β‐catenin signalling‐independent manner, and if so, to delineate the molecular mechanisms.

In the present study, using a well‐established model of striated muscle development,^11^ we discovered a gradual increase in LRP6 protein in the nucleus of differentiating myoblasts, and a positive effect of LRP6 on myotube maturation. The direct functional interactions of LRP6 with the *cis*‐regulatory element in integrin‐β1 (*Itgb1*) pre‐mRNAs and the core spliceosomal components determined the muscle‐specific splicing of *Itgb1D* in striated muscle cells. Moreover, deep RNA sequencing of cells across species highlighted the global splicing regulatory activity of LRP6.

## 2 MATERIALS AND METHODS

### 2.1 Myoblast cell culture and myodifferentiation

The mouse C2C12 myoblasts (CRL‐1772, ATCC®) were grown at low density in Dulbecco's Modified Eagle Medium (DMEM, D6429, Sigma‐Aldrich) supplemented with 10% FBS (F7524, Sigma‐Aldrich), 100 U/mL penicillin, 100 μg/mL streptomycin and 1% L‐glutamine (G7513, Sigma‐Aldrich). After reaching 70% confluence, the medium was replaced by DMEM supplemented with 2% horse serum (26050070, Gibco™) to induce myodifferentiation. The medium was changed every 24 hours before the test. To obtain the knockdown of *Lrp6* in C2C12 cells, adenovirus‐mediated *Lrp6* shRNA that targets the sequence GCACTACATTAGTTCCAAA and control shRNA (ATAGCTACAATCGCAATCT) were transfected into cells. To quantify the myodifferentiation, we calculated the fusion index as the average number of nuclei in MHC‐positive cells with at least three nuclei above total number of nuclei, and the number of nuclei per myotube was measured using the NIH Image J software.

### 2.2 Isolation and culture of neonatal rat cardiomyocytes

The isolation and culture of neonatal rat cardiomyocytes (NRCMs) were performed according to the procedures previously described.^7^

### 2.3 Cellular fractionation

Cytoplasmic and total nuclear proteins were extracted using the CelLyticTM NuCLEARTM Extraction Kit (NXTRACT, Sigma) according to the manufacturer's instructions.

### 2.4 Immunofluorescence microscopy

HeLa cells were transfected with vectors expressing the flag‐fused protein containing the full‐length LRP6 for 48 hours. Cells were fixed with 4% paraformaldehyde for 15 minutes, and permeabilized with 0.5% Triton X‐100 in phosphate‐buffered saline for 10 minutes, following by 30 minutes of blocking with 1% goat serum (S2263, Sigma). The fixed cells were then incubated with mouse monoclonal antibodies, including anti‐flag (1:200, F3165, Sigma), anti‐U2F65 (1 μg/mL, ab37530, Abcam), anti‐CUG‐BP1 (1:100, ab129115, Abcam) and anti‐PTBP1 (1:250, ab133734, Abcam), respectively. The Alexa‐488/555‐conjugated secondary antibodies against mouse or rabbit IgG (1:300, A11001, A‐21428, Molecular Probes) were used. After nuclei were counterstained with 4, 6‐diamidino‐2‐phenylindole (DAPI, 5 μg/mL), the cells were visualized using a Leica confocal laser‐scanning microscope.

### 2.5 mRNA stability assays

*Itgb1D* mRNAs in NRCMs were quantified relative to 18S rRNA at various times after addition of actinomycin D (ActD, 6.5 μg/mL, Sigma‐Aldrich) to the culture medium. Relative quantification values at 0 hour were set to 1.

### 2.6 Protein purification

To obtain the purified proteins of the full‐length LRP6, the vector expressing LRP6 was constructed. The corresponding sequence was amplified from *Lrp6* plasmid (Origene) by PCR and was subcloned into the pcDNA3.1 expression vector (Invitrogen) using BamH1 and Xbal restriction sites. The DNA sequence of the flag tag was attached to the C‐terminus of *Lrp6*. The plasmid was transfected into the 293T cells for 48 hours using Lipofectamine3000 (Invitrogen), and the overexpressed LRP6 proteins were purified using FLAG® M Purification Kit (Sigma).

### 2.7 Biotinylated RNA pull‐down assays

The full‐length U1‐U6 snRNAs (rat) were transcribed in vitro using T7 RNA polymerase (ThermoFisher), and then were labelled using Pierce RNA 3′ end desthiobiotinylation kit (ThermoFisher). 150 pM RNA probes were incubated with 50 μL streptavidin magnetic beads (Sigma) at room temperature for 25 minutes. Then, 400 ng of the purified flag‐tagged LRP6 fusion proteins were incubated with RNA‐beads complex at 4°C for 50 minutes. The proteins bound to U1‐U6 snRNAs were stripped by elution buffer and analysed by Western blotting with anti‐LRP6 antibody (C5C7, CST).

### 2.8 Plasmid construction

To construct recombinant vectors of U1‐U6 snRNAs, their sequences were amplified from rat cDNAs and subcloned into the pCMV6‐entry vector (Origene) using SgfI and XhoI restriction sites. The human *Itgb1* gene luciferase reporter plasmid was constructed through inserting *Itgb1* promoter into pGL3‐basic vector (Promega) using the SacI and XhoI restriction sites. To generate vectors expressing different LRP6 domains including P1E1P2E2, P3E3P4E4 and ICD, the corresponding sequences were amplified from *Lrp6* plasmid (Origene) and subcloned into the pcDNA3.1 expression vector (Invitrogen) using BamHI and XbaI restriction sites. To construct the NLS‐GFP expression vector, the triple nuclear localization signal (NLS) sequences were synthesized (Sangon Biotech, Shanghai) and inserted into multi‐clone site of pEGFP‐N1 using SacI and AgeI restriction sites. The wild ICD and mutated ICD sequences (Sangon Biotech, ShangHai) were inserted into the carboxy terminus of GFP to construct NLS‐GFP‐ICD^Wt^/ICD^Mut^ through homologous recombination. All constructs were confirmed by DNA sequencing. All standard PCR reactions were performed with KOD polymerase (TOYOBO), and the sequences of all PCR primers were provided in Table S1.

### 2.9 Minigene constructs and site‐directed oligonucleotide mutation

The minigene constructs were assembled in the pCMV6‐entry vector (Origene). The sequences of alternative exon D within human *Itgb1* gene pre‐mRNA and the flanking introns were amplified from human genomic DNA. *Itgb1* minigene variants were generated by replacing the alternative exon D or the flanking introns with the mutated sequences. The constructs containing the serial deletions (~10 bp) within alternative exon D were created by overlapping PCR mutagenesis using primers suitable for *Itgb1*. The identity of each minigene was verified by sequencing analysis. Single nucleotide substitutions were introduced in alternative exon D by overlapping extension PCR. The sequences of all PCR primers were provided in Table S1.

### 2.10 Luciferase assays

To measure the transcription activity of *Itgb1* gene, pGL3‐*Itgb1* promoter luciferase reporter vector and pRL‐TK (Promega) were co‐transfected in the HeLa cells, which were subject to *Lrp6* silencing by the targeted siRNAs for 48 hours. After another 24 hours, cells were harvested and analysed using a Dual‐Luciferase reporter assay kit (Promega).

### 2.11 Immunoblotting analysis

Proteins were extracted on ice using a RIPA lysis buffer (50 mM Tris (pH 7.4), 150 mM NaCl, 1% NP‐40, 0.5% sodium deoxycholate, 0.1% SDS) supplemented with protease inhibitor‐cocktail (Roche). SDS‐PAGE and western bolt were performed to analyse the expression of target proteins according to standard protocols. Primary antibodies in western blotting examination included the following: myogenin (ab1835, Abcam), MHC (ab180779, Abcam), GAPDH (D16H11, CST), ITGB1 (ab179471, Abcam), ITGB1D (97733, NOVUS), ITGB1D (ab8991, Abcam), LRP6 (C5C7, CST), α‐Tubulin (1124‐1‐AP, Proteintech), Histone H3 (ab1791, Abcam), GFP (2555, CST), hnRNP A1 (ab123378, Abcam), PTBP1 (ab133734, Abcam), β‐catenin (C2206, Sigma). The secondary antibodies conjugated to infrared dyes (LI‐COR Biosciences) were applied at a concentration of 1:10 000, and the blots were visualized using an Odyssey imager (LI‐COR Biosciences).

### 2.12 In vivo splicing assay

HeLa cells and NRCMs were transfected with either plasmids expressing the NLS‐fused proteins including NLS‐GFP (0.75 and 1.5 μg), NLS‐GFP‐ICD (0.75 and 1.5 μg) and NLS‐GFP‐ICD mutant (1.5 μg), respectively, or *Lrp6* siRNAs using RNAiMAX (Invitrogen). Twenty‐four hours later, *Itgb1*‐minigene reporters were overexpressed for another 24 hours in these cells with Lipofectamine 3000 (Invitrogen). Cellular RNAs were extracted using TRI reagent (Ambion) and then treated with RNase‐free DNase I (Promega) for 30 minutes at 37°C. Complementary DNA was generated by reverse transcription with a poly T primer for 30 minutes at 37°C using reverse transcriptase (Takara). Semi‐quantitative RT‐PCR was performed through GoTaq® Green Master Mix (promega) on a PCR system (Applied Biosystems), and the products were identified by agarose gel electrophoresis.

### 2.13 RNA immunoprecipitation

To analyse the interaction of LRP6 protein with *Itgb1* mRNA and the interaction of LRP6 domains with *Itgb1D* mRNA, the pCMV6‐entry‐pre‐*Itgb1*, pCMV6‐entry*‐Itgb1D* and pcDNA3.1‐Flag‐*Lrp6*‐P1E1P2E2/P3E3P4E4/ICD expression plasmids were, respectively, transfected into NRCMs with lipofectamine 3000 for 48 hours. Following the constructions of the EZ‐Magna RIP™ RNA‐Bing Protein Immunoprecipitation Kit (Millipore), RNAs were extracted and analysed by RT‐PCR and agarose gel electrophoresis. The assay kit was also used to measure the interaction of endogenous LRP6 protein and U1/2/4/5/6 snRNAs.

### 2.14 RNA sequencing and alternative splicing detection

Paired‐end sequencing was performed on an Illumina hisseq4000 (GEO access number: GSE102770). Reads were mapped to the human or rat genome assembly using Tophat (http://tophat.cbcb.umd.edu/) (version 2.0.9). Aligned reads were then quantified for expression using the Cufflinks suite version 2.1.1 (http://cufflinks.cbcb.umd.edu/) and edgeR (http://www.bioconductor.org/packages/release/bioc/html/edgeR.html). The alternative splicing detector (ASD, available on http://www.novelbio.com/asd/ASD.html) was selected as the tool to detect the differentially alternative splicing cases based on the bam file after mapping and reference genome sequencing, according to the adjusted P value threshold (adjusted *P* value <.05).

### 2.15 Statistical analysis

All data are presented as the means ± standard errors of the mean (SEM). The data shown were the averages of at least three biological replicates. No inclusion/exclusion criteria were used. Statistical analyses were performed using Prism Software (GraphPad). The statistical significance of the difference between two sets of data was assessed using an unpaired, two‐tailed Student's *t* test and one‐way anova with Bonferroni's post‐hoc test. A *P* value less than .05 was considered to be significant.

## 3 RESULTS

### 3.1 LRP6‐mediated muscle development couples to the muscle‐specific splicing of *Itgb1D*

**Figure 1:**
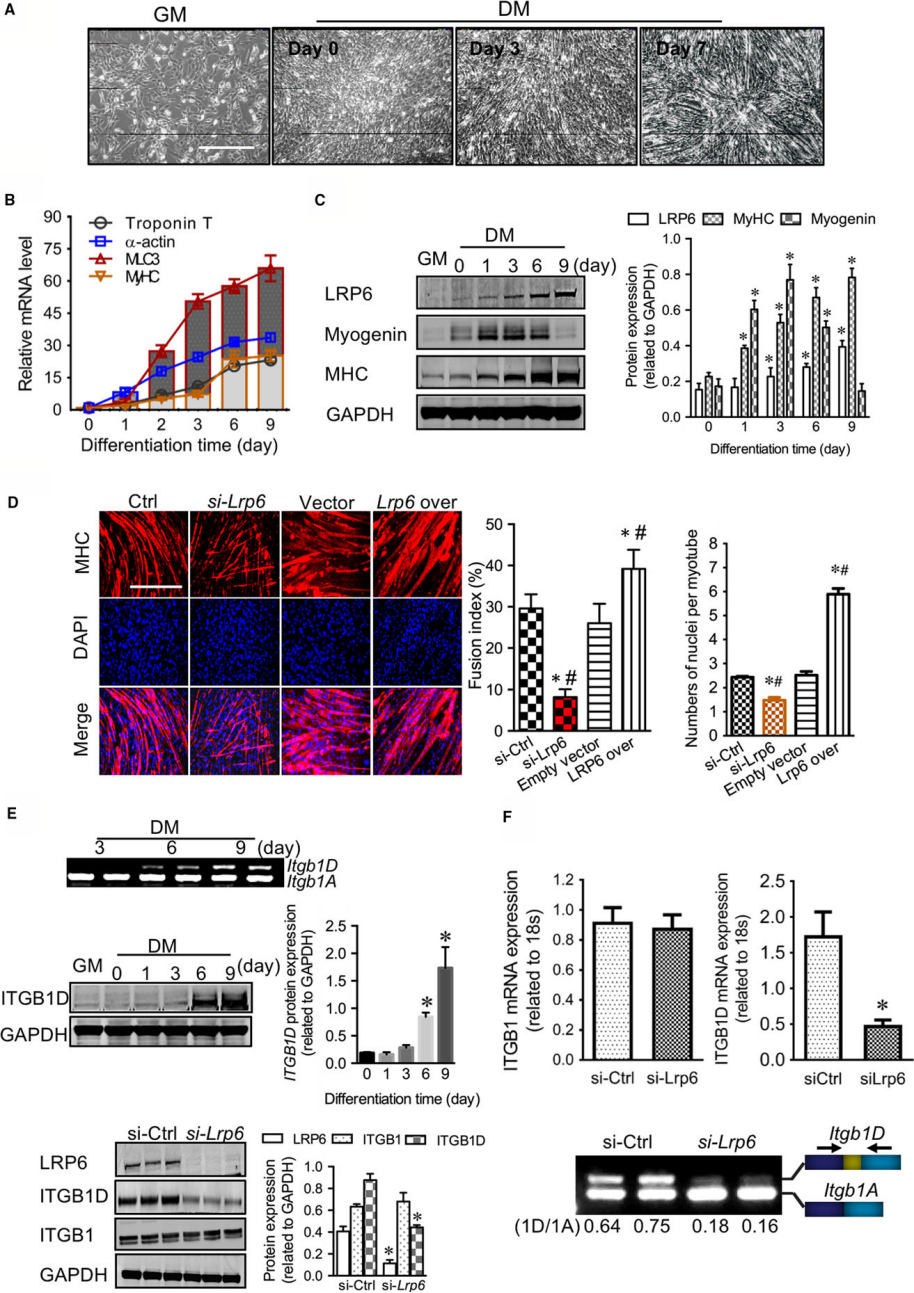
LRP6 regulates the muscle‐specific splicing of integrin‐β1D during the development of striated muscle. (A) Time‐lapse microscopy of C2C12 myoblasts throughout the proliferation and myodifferentiation phases. GM: growth medium; DM: differentiation medium. Scale bar: 10 μm. Quantitative PCR (B) and Western blotting (C) analysis of LRP6 and myodifferentiation markers at indicated time points. C‐left: typical blots; C‐right: pooled data. *P < .05 compared with Ctrl, ^#^ *P* < .05 compared with groups other than Ctrl. (E) Measurement of integrin‐β1 transcripts and proteins in C2C12 cells. Upper: examination of integrin‐β1 transcripts during myodifferentiation using gel electrophoresis; Middle and Lower: Western blotting examination of integrin‐β1 (ITGB1) and ITGB1D during myodifferentiation (middle) and under *Lrp6*‐deficient conditions (lower). E‐middle and ‐lower left: typical blots; E‐middle and ‐lower right: pooled data. *P < .05 compared with Ctrl. Representative images from five independent experiments with similar results are shown

To test whether LRP6 modulates tissue development and homeostasis in a Wnt signalling‐independent manner, we utilized a C2C12 myoblast model that recapitulates important features of striated muscle development. These myoblasts underwent a program of proliferation and myodifferentiation to form multinucleated myotubes (Figure 1A,B). During the proliferative phase, LRP6 protein was barely detectable in C2C12 myoblasts. Its expression prominently increased when myoblasts entered into myodifferentiation, and peaked when myotube formed (Figure 1C). The coincidence of the onset of LRP6 expression with the timing of myoblast withdrawal from the cell cycle implied a major function for LRP6 in commitment of differentiating muscle cells, which was evidenced by the findings that LRP6 deficiency greatly retarded, whereas its overexpression promoted, the myotube fusion (Figure 1D).

During striated muscle development, integrin‐dependent cell‐matrix adhesion sets the starting point for myofibrillogenesis.^12^ Different splicing isoforms of *Itgb1* are differentially expressed in foetal (*Itgb1A*) and mature muscle cells (*Itgb1D*), and the latter is essential for myotube fusion.^13^, ^14^ We found that ITGB1D protein was gradually increased in parallel with LRP6 protein during myodifferentiation (Figure 1E). LRP6 deficiency down‐regulated the expression of ITGB1D protein, but did not affect the total ITGB1 proteins (Figure 1E). The reduction of ITGB1D proteins was not involved in the transcriptional change of *Itgb1* and the degradation of *Itgb1D* mRNA and protein (Figure S1 and S2). However, while the overall amount of *Itgb1* mRNAs appeared normal, the transcript levels of *Itgb1D* were decreased and concurrently the expression of *Itgb1A* mRNAs up‐regulated by LRP6 deficiency, suggesting the deregulated splicing switching of *Itgb1A* to muscle‐specific *Itgb1D* isoforms (Figure 1F). These evidence indicate that LRP6 may act as a trans‐acting regulator mediating the functional splicing of *Itgb1* pre‐mRNAs.

### 3.2 LRP6 regulates the muscle‐specific splicing of *Itgb1D* independently of the Wnt/β‐catenin signalling

LRP6 directs the development of multiple tissues putatively through the Wnt signalling mediated by β‐catenin, which translocates to the nucleus and interacts with transcriptional activators to modulate the transcription of target genes that control cell cycle, proliferation and growth.^2^ However, we observed that β‐catenin proteins remain relatively constant throughout the processes of proliferation and myodifferentiation (Figure S3A), in sharp contrast to the gradual increase in LRP6 protein. Moreover, β‐catenin negatively regulated the transcription of *Itgb1* to affect ITGB1D expression and myotube fusion (Figure S3B‐E), as opposed to the effects mediated by LRP6. This scenario implicated that the LRP6‐mediated muscle‐specific splicing of *Itgb1D* is independent of Wnt/β‐catenin signalling, but LRP6 played an antagonistic role to the β‐catenin in the expression of *Itgb1D* isoform.

### 3.3 LRP6 interacts with the core spliceosome components to direct muscle‐specific splicing of *Itgb1D* in the nucleus of striated muscle cells

**Figure 2:**
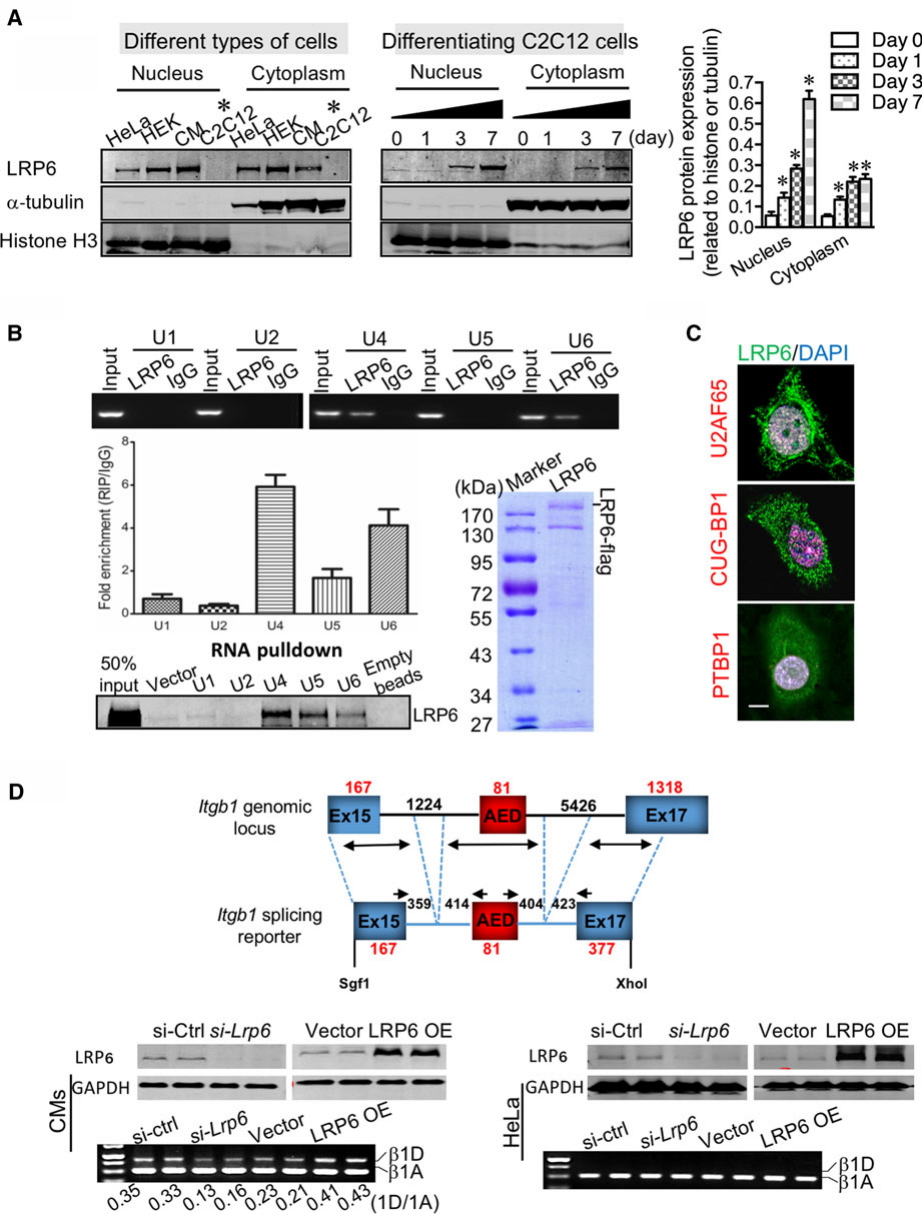
Nuclear LRP6 directs the muscle‐specific splicing of *Itgb1D* in striated muscle cells. (A) Identification of LRP6 in the nuclei of cells. CM: neonatal rat cardiomyocytes. The α‐tubulin and histone H3 were used as cytoplasmic and nuclear markers, respectively. Right: pooled data. *P < .05 compared with Day 0. (B) Binding of LRP6 protein to small nuclear RNAs (U1/2/4/5/6 snRNAs), as detected by immunoprecipitation from lysates of CM cells (left‐upper and ‐middle) and an in vitro protein‐RNA pull‐down assay with the purified LRP6 proteins and small nuclear RNAs (U1/2/4/5/6 snRNAs) (left‐lower). Right, Coomassie blue staining of the purified LRP6‐flag recombinant proteins. (C) In the nuclei of HeLa cells, LRP6 partially co‐localized with the splice factors U2AF65, PTBP1 and CUG‐BP1. Scale bar, 5 μm. (D) Specific activity of LRP6 on *Itgb1* RNA monitored in a cell‐based splice‐reporter minigene assay using the exons that encode the alternative exon D. Top, schematic representation of the *Itgb1* splicing reporter. Western blotting examination of LRP6 protein and gel electrophoresis of *Itgb1* RNAs were conducted in the neonatal cardiomyocytes (CMs) (Bottom‐left) and the HeLa cells (Bottom‐right) subject to LRP6 knockdown and overexpression. Representative images from five independent experiments with similar results are shown

As the splicing regulation is well characterized in the eukaryotic nucleus,^15^ we expected that LRP6 is physiologically present at appreciable levels in the nucleus. Unexpectedly, LRP6 protein was almost undetectable in the nucleus of C2C12 myoblasts. However, as soon as myodifferentiation commenced, the nuclear expression of LRP6 was dramatically enhanced (Figure 2A), which was positively pertinent to the degree of myodifferentiation. Interestingly, the nuclear expression of LRP6 protein was constantly detected in some other types of cells, including HeLa, HEK293 and cardiomyocytes (Figure 2A).

Nuclear pre‐mRNA splicing is carried out in the spliceosome, a dynamic RNA‐protein complex composed of five small nuclear RNAs (U1/2/4/5/6 snRNAs) together with associated splice factors.^16^ At the initial step of splicing, U1 and the U2 auxiliary factors (U2AF) bind to the 5′ and 3′ splice site, respectively. Successively, U2 tethers to the branch point, and a preformed complex of U4/5/6 tri‐snRNPs is recruited to the intron. After release of the U1 and U4, the splicing reaction is catalysed. Using RNA‐immunoprecipitation (RIP) and in vitro biotinylated RNA pull‐down assays, we identified that LRP6 bond to U4/5/6 snRNAs (Figure 2B). In addition, the immunofluorescent staining also revealed the partial co‐localization of endogenous LRP6 protein with the splice factors U2AF65, C1 and PTBP1 in the nucleus of HeLa cells (Figure 2C). The U2AF65 protein contains a serine/arginine (SR)‐rich domain that recruits itself to active splicing sites.^17^, ^18^ These results suggest that LRP6 may function at the catalytic step of splicing.

Moreover, the specific activity of LRP6 on *Itgb1* pre‐mRNA was confirmed using a splicing reporter minigene in which exclusion of the alternative exon D (AED) contributes to *Itgb1*'s elasticity (Figure 2D). Considering the case that the efficacy of the minigene expression was low in C2C12 cells, we conducted the activity analysis of the splicing reporter in intact cultured neonatal rat cardiomyocytes that share the same expression profiles of *Itgb1A/1D* isoforms. Reduction of LRP6 induced the exclusion of AED in neonatal rat cardiomyocytes; however, LRP6 protein did not affect the usage of AED in HeLa cells, indicating that LRP6‐mediated *Itgb1* splicing is muscle‐specific and additional splicing cofactors missing in non‐muscle cells are necessary for the splicing process.

### 3.4 The C‐terminal serine‐rich region in LRP6 directly targets the adenine‐rich sequence within AED to determine the usage of AED

**Figure 3:**
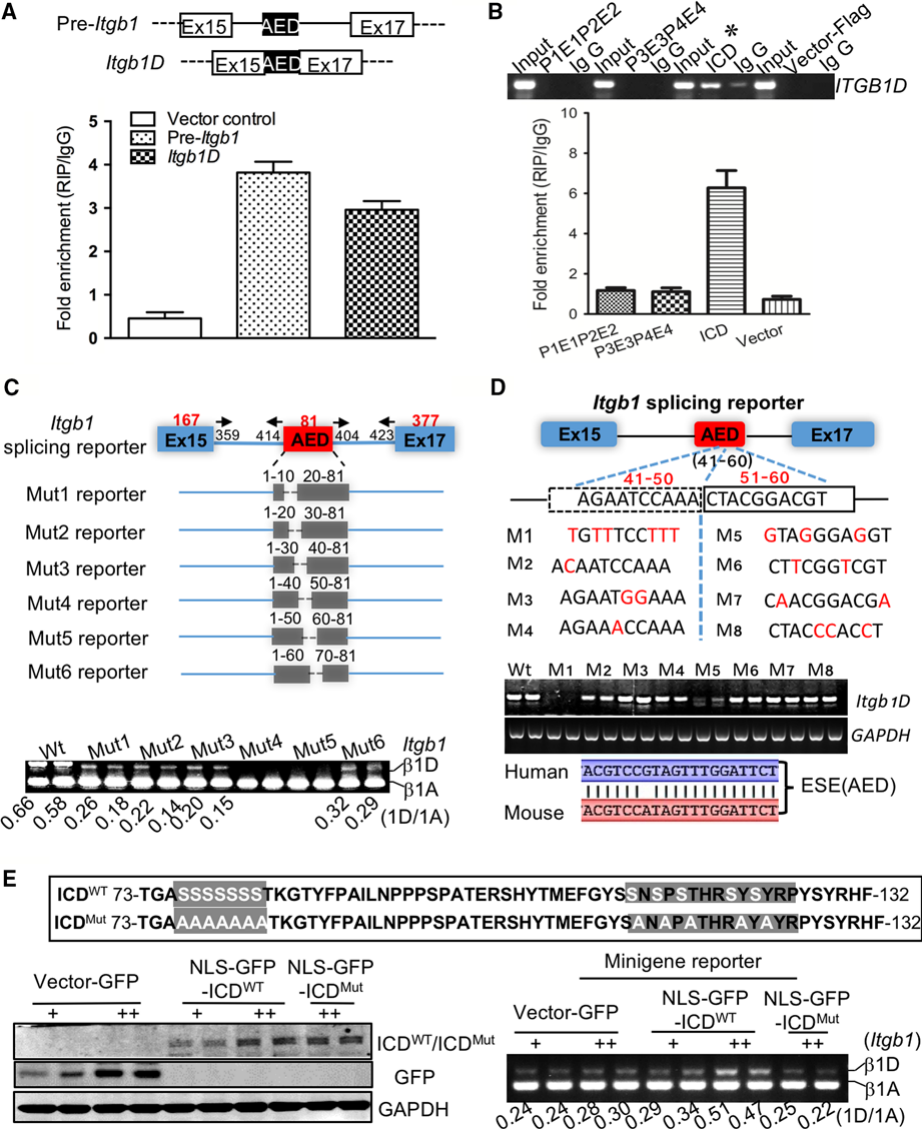
Interaction of LRP6 with exon splicing enhancer determines the muscle‐specific splicing of *Itgb1D*. (A) Quantitative PCR detection of precursor *Itgb1* and mature *Itgb1D* mRNAs retrieved by LRP6‐specific antibody compared with immunoglobulin G (IgG) in the RIP assay within NRCMs cells transfected with vectors expressing pre‐*Itgb1* and *Itgb1D* mRNAs, respectively. (B) Identification of the LRP6 modules responsible for *Itgb1D* RNA binding. Gel electrophoresis (Top) and qPCR analysis (bottom) of *Itgb1D* RNAs immunoprecipitated by the LRP6 domains fused to the Flag tag. (C) In vivo selection of exonic splicing enhancer sequences. Top, schematic representation of serial deletions of the 81‐bp Exon D in the splicing reporter minigene construct; bottom, gel electrophoresis analysis of *Itgb1* RNAs extracted from neonatal cardiomyocytes expressing NLS‐ICD and minigene mutants. Mut: mutation. Wt: wild type. (D) A point mutation‐dependent strategy utilized to visualize the cis‐regulatory element. Upper, diagram showing the point mutation; middle, gel electrophoresis analysis of *Itgb1* RNAs extracted from neonatal cardiomyocytes expressing NLS‐ICD and minigene mutants. M: mutation. Lower, analysis of the identity of ESE in AED between mouse and human. (E) Identification of the amino acid residues responsible for LRP6 binding to the exonic splicing enhancer. Top, Diagram showing the point mutation within LRP6 C‐terminus. Bottom‐left, western blotting verification of the expression of wild and mutated NLS‐ICD (NLS‐GFP‐ICDWT and NLS‐GFPICDMut) proteins in cultured neonatal cardiomyocytes. Bottom‐right, Gel electrophoresis analysis of *Itgb1* RNAs. Representative blots from five independent experiments with similar results are shown. Mut, mutation. WT, wild type

The exclusion or inclusion of alternative exons highly depends on the position and context of splicing *cis*‐regulatory sequences within alternative exons or the flanking introns, including exonic splicing enhancers or silencers (ESEs or ESSs) and intronic splicing enhancers or silencers (ISEs or ISSs) that recruit splicing regulators.^16^, ^17^ Using the RIP approach, we observed that both mature *Itgb1D* mRNA and *Itgb1* pre‐mRNA, which share the 81‐bp AED sequence, were detected in LRP6 protein‐immunoprecipitated transcripts (Figure 3A), implicating that the AED was the potential *cis*‐regulatory element. Furthermore, the *Itgb1D* RNAs were identified in the transcripts immunoprecipitated by proteins derived from the intracellular carboxy‐terminal domain (ICD) rather than other modules of LRP6 (Figure 3B), suggesting LRP6‐ICD as a RNA‐recognition motif.

To examine, in more detail, the characteristics of RNA elements responsible for the AED usage, we introduced serial deletions of the 81‐bp AED into the minigene construct and analysed the effects of each mutant on the inclusion of AED in vivo. As shown in Figure 3C, expression of mutant minigenes containing the deletion of the 20‐bp between 41 and 60 base pairs showed the significant exclusion of AED, suggesting that the 20‐bp fragment has LRP6‐dependent enhancer activity. Using a point mutation‐dependent strategy, we further identified that the mutation of A to T in the 10‐bp sequence between 41 and 50 bp almost completely depressed the AED inclusion (Figure 3D), which was identical to the effects by 20‐bp deletion, whereas the mutation of C to G between 51 and 60 bp partially weakened the AED inclusion, implicating the adenine‐rich sequence within AED as the core ESE.

Next, we defined the amino acid resides responsible for targeting the ESE in AED. The web‐based RNA‐binding analysis showed that the 73‐132 amino acid regions within LRP6‐ICD domain are highly preferential for RNA binding (Figure 3E), in which the serine is rich. Using the site‐directed mutagenesis, we identified that the nuclear leading sequence (NLS)‐mediated expression of the ICD mutant containing a Ser‐to‐Ala substitution (NLS‐ICDMut) induced the exclusion of AED in the neonatal cardiomyocytes expressing wild‐type minigene (Figure 3E), indicating that the serine residue was critical for the RNA splicing activity. Overall, LRP6 may act primarily as a splicing regulator to facilitate the inclusion of AED within *Itgb1* pre‐mRNA in striated muscle cells.

### 3.5 LRP6‐mediated AED use is sufficient to overcome exon skipping by splicing repressors

**Figure 4:**
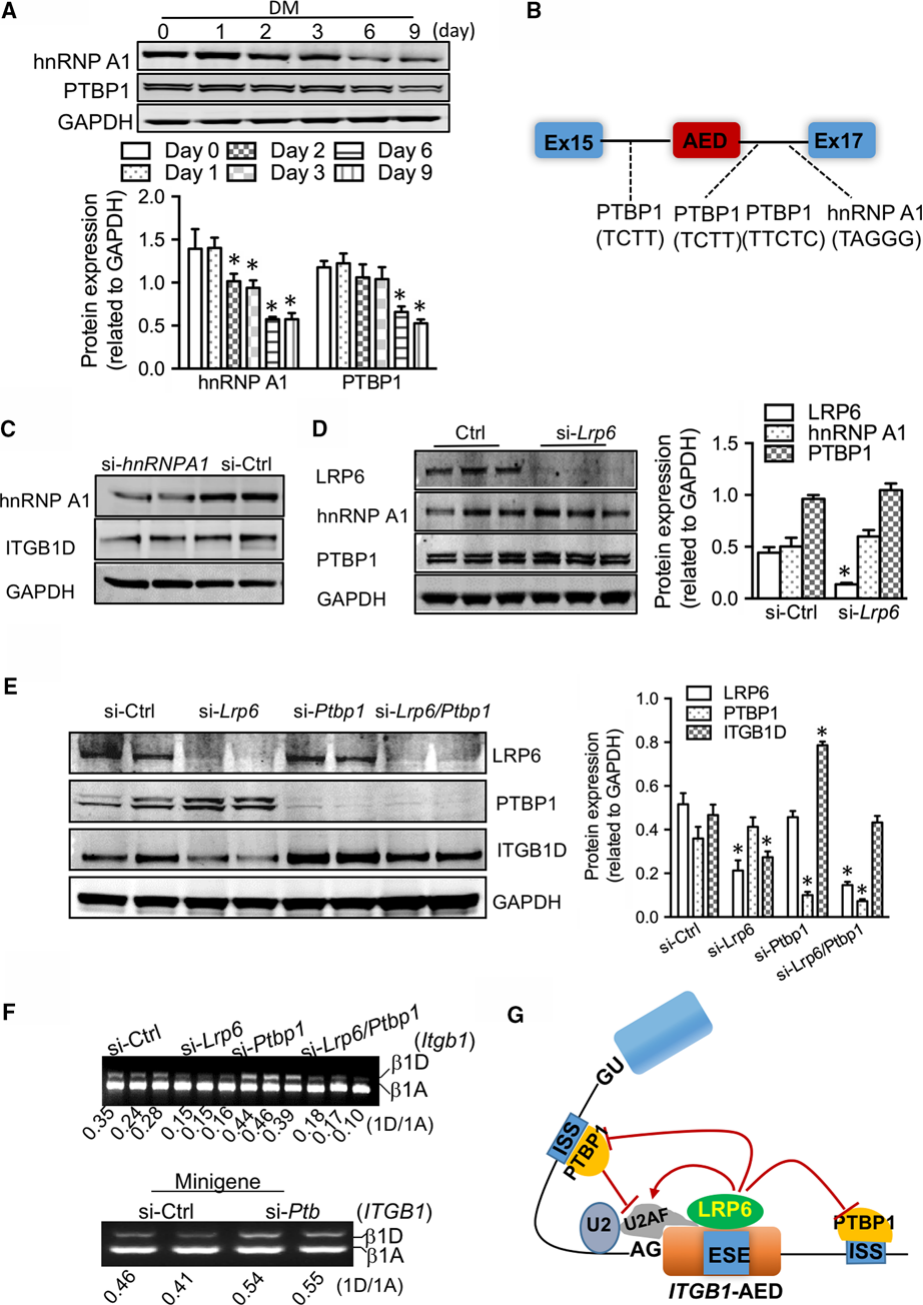
Roles of splicing repressors in the splicing switching of *Itgb1* isoforms. (A) Western blotting examination of hnRNP A1 and PTB proteins during myodifferentiation of C2C12 myoblasts. DM, differentiation medium. Bottom: pooled data. *P < .05 compared with day 0. (B) Schematic of hnRNP A1 and PTB‐binding sites in the flanking introns of alternative exon D in the splicing reporter of *Itgb1*. (C) Effects of hnRNP A1 on the protein expression of ITGB1D in myocytes. (D) Effects of LRP6 on the protein expression of hnRNP A1 and PTBP1. The proteins were extracted from neonatal cardiomyocytes subject to *hnRNP A1*‐ or *Lrp6*‐siRNAs for 48 h. Right: pooled data. *P < .05 compared with si‐Ctrl. (E‐F) Effects of the splice repressor PTBP1 on the splicing of *Itgb1D* in the presence and absence of LRP6 in culture neonatal cardiomyocytes. Western blotting examination of LRP6, PTBP1 and ITGB1D proteins (E). E‐right: pooled data. *P < .05 compared with si‐Ctrl. (F‐top) Gel electrophoresis analysis of *Itgb1* RNAs; (F‐bottom) gel electrophoresis analysis of the specific activity of PTBP1 on *Itgb1* pre‐mRNA using the minigene. (G) Model of the LRP6‐mediated splicing of *Itgb1D* in striated muscle cells. Representative blots from five independent experiments with similar results are shown

The usage of a particular exon is usually subject to antagonistic factors‐mediated bidirectional regulation.^17^ Members of the hnRNP family often cause exon exclusion through binding to splicing *cis*‐regulatory elements.^18^ During myodifferentiation, the hnRNP A1 (also known as hnRNP I) and PTBP1 exhibited the expression pattern opposite to LRP6 protein (Figure 4A). Examination of the splicing minigene revealed the presence of binding sites for hnRNP A1 and PTBP1 in the flanking introns of AED (Figure 4B). Reduction of hnRNP A1 did not affect the inclusion of AED in neonatal cardiomyocytes (Figure 4C), and LRP6 did not affect the protein expression of hnRNP A1 and PTBP1 (Figure 4D). Although PTBP1 reduction promoted the inclusion of AED, the LRP6 deficiency‐induced AED exclusion was greatly counteracted by PTBP1 down‐regulation (Figure 4E,F), indicating that targeting of LRP6 to ESE within AED was sufficient to overcome the AED exclusion by PTBP1 splicing repressor (Figure 4G), achieving the splicing of *Itgb1D* isoform.

### 3.6 LRP6 guides the global regulation of AS

**Figure 5:**
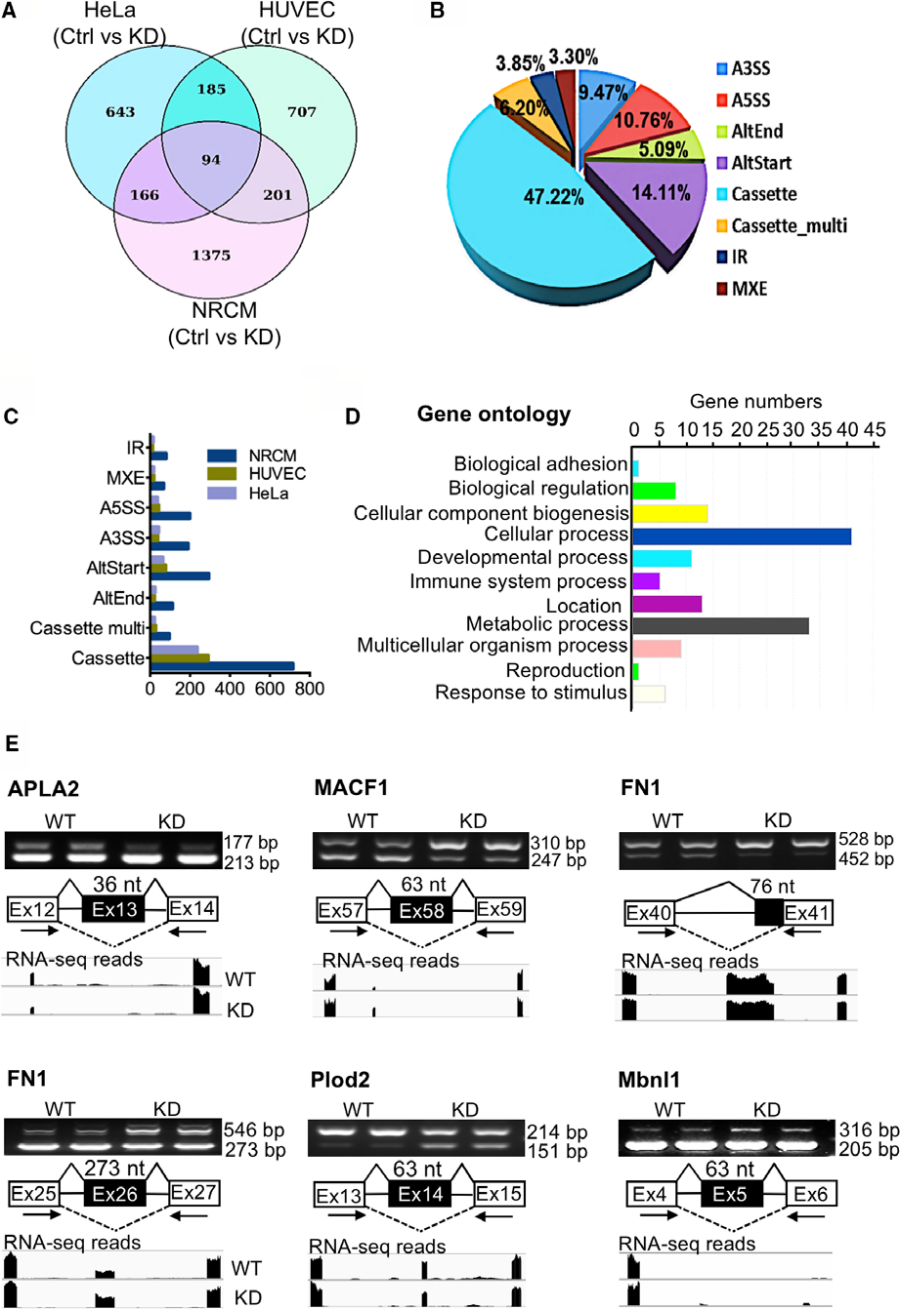
LRP6 guides the global RNA splicing regulation. (A‐C) Genome‐wide identification by RNA‐seq of *Lrp6* loss‐induced alternative splicing (AS) events in cardiomyocytes (NRCM), HeLa cells and human umbilical vein endothelial cells (HUVEC). (A) Venn diagram for the differential AS events in wild type (Ctrl) and *Lrp6* knockdown (KD) cells; (B) The pie chart for the percentage of different types of the differential AS events; (C) Number of total LRP6‐dependent AS events. (D) Functional categories of a conserved set of 94 genes with LRP6‐dependent AS between humans and rats. (E) The AS of genes identified by RNA‐seq was verified through RT‐PCR analysis in NRCMs. WT, wild type; KD,* Lrp6* knockdown

To determine whether the LRP6‐mediated splicing regulation is a more widespread phenomenon, we performed deep RNA sequencing of cells from rat and human. We detected a substantial number of *Lrp6* loss‐induced alternative splicing events, and up to nearly 50% of them were exon‐skipped (Figure 5A‐C). This LRP6‐dependent gene network includes *Itgb1*, whose alternative splicing was expected based on the above experiments and thus validated our approach. A gene ontology analysis of the conserved LRP6‐driven network showed enrichment for 94 genes encoding proteins mainly involved in cellular and metabolic processes (Figure 5D). Among them, a decade of genes with LRP6‐dependent isoform expression showed identical splicing patterns on the exon level across species (Table S2). Moreover, we identified that among the 94 genes that are regulated by LRP6 in the three types of cells, only two genes are involved in the Wnt/β‐catenin signalling pathway, and 92 genes are not associated with this signalling (Table S2). The RNA sequencing results were verified for several additional genes other than *Itgb1* (Figure 5E). All of these data supported the notion that LRP6 guides the global regulation of alternative splicing.

## 4 DISCUSSION

Herein, a combination of studies on muscle development model and deep sequencing of cellular transcriptome across species unveiled an unexpected role of LRP6 in the regulation of alternative splicing. First, LRP6 protein in the nucleus of myodifferentiating myoblasts was gradually increased in parallel with the muscle‐specific splicing of *Itgb1D*. Second, the C‐terminal serine‐rich region of LRP6 served as a RNA‐recognition motif to bind the adenine‐rich *cis*‐regulatory element within AED of *Itgb1* pre‐mRNA. Finally, a deep RNA sequencing revealed the LRP6‐dependent RNA splicing in different types of cells. Therefore, we proposed that LRP6 acts as a potential splicing regulator mediating the global regulation on alternative splicing.

Differential pre‐mRNA splicing stands out as a particularly powerful yet subtle mediator for an organism to achieve gene regulation.^19^ The process requires multiple interactions between pre‐mRNAs, small nuclear ribonucleoproteins and splicing factors. Regulation of this process is highly complicated, depending on loosely defined *cis*‐acting regulatory sequence elements, trans‐acting protein factors and cellular responses to varying environmental conditions. Deciphering the splicing code requires understanding of splicing regulatory RNA‐binding proteins (RBPs) and their cis‐acting binding sites.^16^ There are virtually no data on the sequence preferences of RBPs in most organisms. Predictions of splicing regulatory RBPs from sequence features and tissue splicing data have been limited by the fact that the same genomic sequences are recognized differently by a given RBP in different cell types, leading to only a fraction of RBPs studied.^20^ Thus, the unexpected finding of the splicing regulatory RNA‐binding landscape of LRP6 may extend our understanding of alternative splicing regulation.

The interpretation of the regulation of RBPs on a given RNA target exceedingly depends on the cell type. This scenario is clearly observed in the splicing regulation of LRP6 on *Itgb1D*, which occurred in striated muscle cells including myodifferentiating myoblasts and cardiomyocytes. In addition, splicing is highly variable as mRNA‐RBP interactions are transient and of relatively low specificity. Regulatory RBPs must function in conjunction with specific components of the core splicing machinery to guide splice site selection and pairing. Classic trans‐acting splicing regulators are SR proteins (characteristic arginine‐/serine‐rich domains at the C‐terminal end of SR proteins) and hnRNPs.^17^ In general, these SR proteins have a binding preference for purine‐rich exonic sequences.^17^ Interestingly, we identified the interactions of LRP6 with the core spliceosomal components (Figure 2). Taking advantage of site‐directed mutagenesis, we also uncovered the RNA‐recognition motif characterized by serine enrichment within LRP6‐ICD region, and dissected the regulatory RNA elements as purine‐rich sequences in AED of *Itgb1* pre‐mRNA (Figure 3). Given a high degree of biological resemblance to the well‐known SR proteins, LRP6 may be termed as a new type of RBP.

Pre‐mRNA splicing is conditionally regulated by signal transduction pathways.^16^ Although LRP6 protein has long been thought to mediate the Wnt/β‐catenin signalling, our recent findings indicated that LRP6 is not necessary for transducing Wnt signalling in striated muscle cells.^7^ Especially, despite the soluble LRP6‐ICD has the potential to modulate the activity of β‐catenin‐coupled transcription factor TCF/LEF‐1, affecting the transcription of target genes,^9^ we did not observe the effect of LRP6 on *Itgb1* transcription (Figure 1 and Figure S1). Instead, the downstream effector β‐catenin of the Wnt signalling demonstrated a transcriptional regulation on *Itgb1D* production (Figure S3). Additionally, LRP6 had no effects on the expression of β‐catenin proteins in myocytes. Thus, in the case the splicing of *Itgb1D*, LRP6 works in the Wnt/β‐catenin signalling‐independent manner. However, as far as the control of *Itgb1D* expression is concerned, LRP6 may exert an effect antagonistic to the Wnt/β‐catenin signalling.

In sum, our findings identify LRP6 as a potential splicing regulator. The RNA‐binding landscape of LRP6 may also provide insights into the post‐transcriptional RNA processing, given that RBPs can modulate diverse post‐transcriptional processes including mRNA transport, localization, stability and translation, and microRNA inhibition.^21^ In addition, the disease‐relevant mutations of *Lrp6* have been reported in its splicing regulatory C‐terminus,^2^ and thus there remain great interests to identify whether and how LRP6 affect the potential splicing events linked to tissue and organ pathophysiology.

## CONFLICTS OF INTEREST

The authors declare that they have no conflicts of interest with the contents of this article.

## ACCESSION NUMBER

RNA‐seq data have been deposited in GEO under accession number: GSE102770.

## AUTHOR CONTRIBUTIONS

Y.‐H.C. and J.L designed the study, J.L., T.Y., S.W. and C.H. performed the majority of experiments, J.L., T.Y. and S.W. analysed the data. T.Y., D.L., Y.W., X.L., L.L and Y.L performed the antibody immunofluorescence, site‐mutagenesis, cell culture and the generation of wild and mutated minigene reporters. T.Y. assisted in RT‐PCR splicing assays. J.L., S.W., C.H., Y.W. and T.Y. performed RNA‐seq experiments. Y.‐H.C. and J.L. wrote the manuscript.
