# Supplementary material for: Low‐density lipoprotein receptor‐related protein 6 regulates alternative pre‐mRNA splicing
Source: J Cell Mol Med. 2018 Aug 1;22(10):4653–63. doi: 10.1111/jcmm.13682 (PMC6156287; doi:10.1111/jcmm.13682)

**
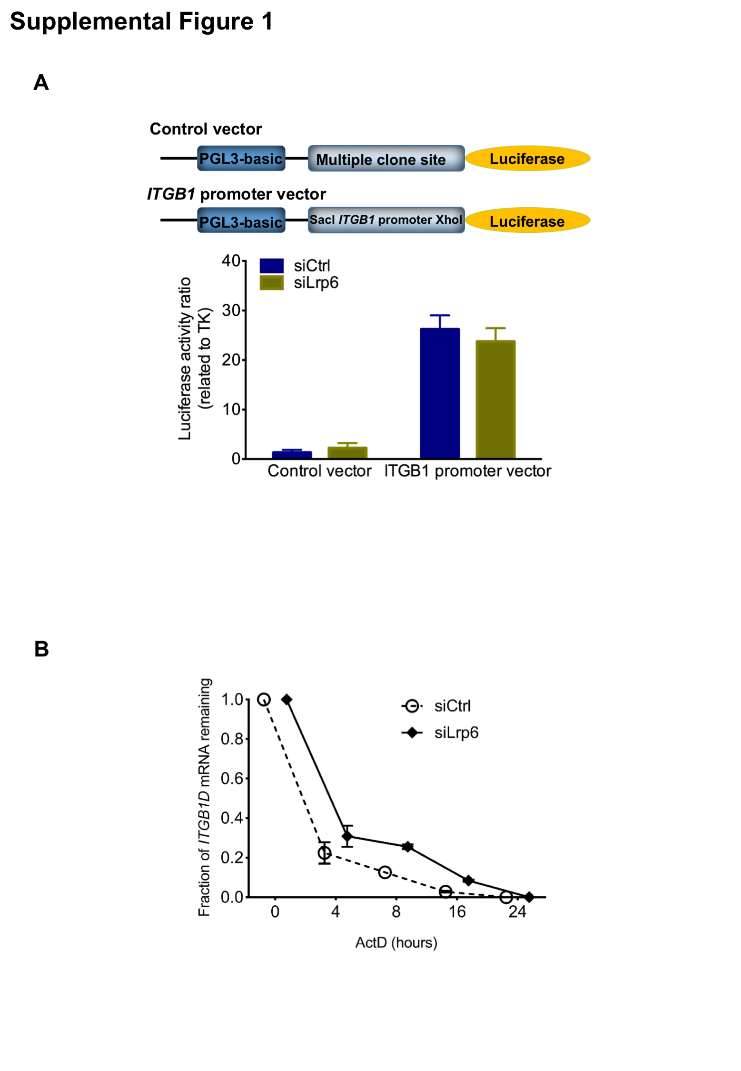
**

**Fig. S1.** LRP6 does not affect ITGB1 transcription and ITGB1D mRNA degradation. (A) Luciferase assay for the transcription of ITGB1. Top, schematic diagram of the luciferase report vector containing the ITGB1 promoter; bottom, pooled data. LRP6-deficient HeLa cells were transfected with a ITGB1 promoter reporter construct for 24 h before activity measurement. (B) The decay of ITGB1D mRNA was determined in Lrp6-deficient neonatal cardiomyocytes. Relative quantitative values at 0 h were set to 1. The presented data from three to five independent experiments with similar results are shown.


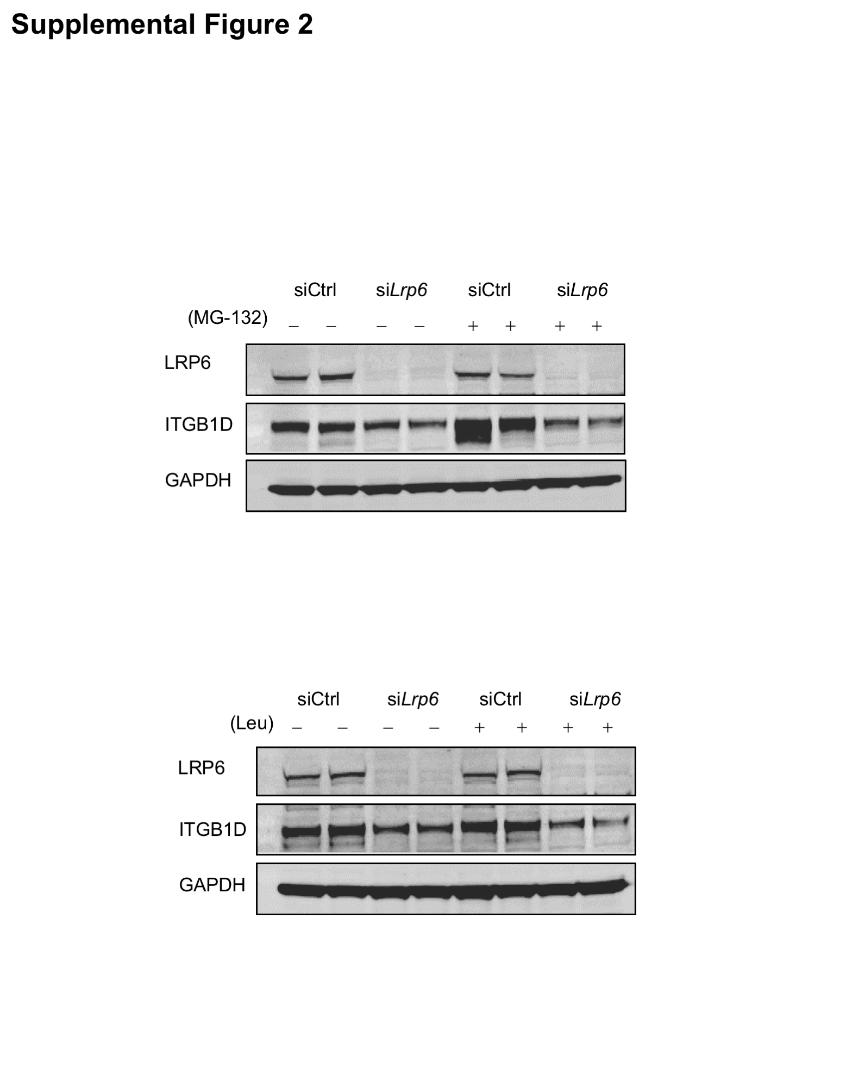


**Fig. S2.** LRP6 does not affect the stability of ITGB1D protein. In neonatal cardiomyocytes subject to Lrp6 siRNAs for 48 hours, treatment with MG-132 (a proteasomal inhibitor, 0.5 μM) and Leu (a lysosomal inhibitor, 100 μM) for another 24 hours did not counteract the reduction of ITGB1D protein. Representative blots from three independent experiments with similar results are shown.


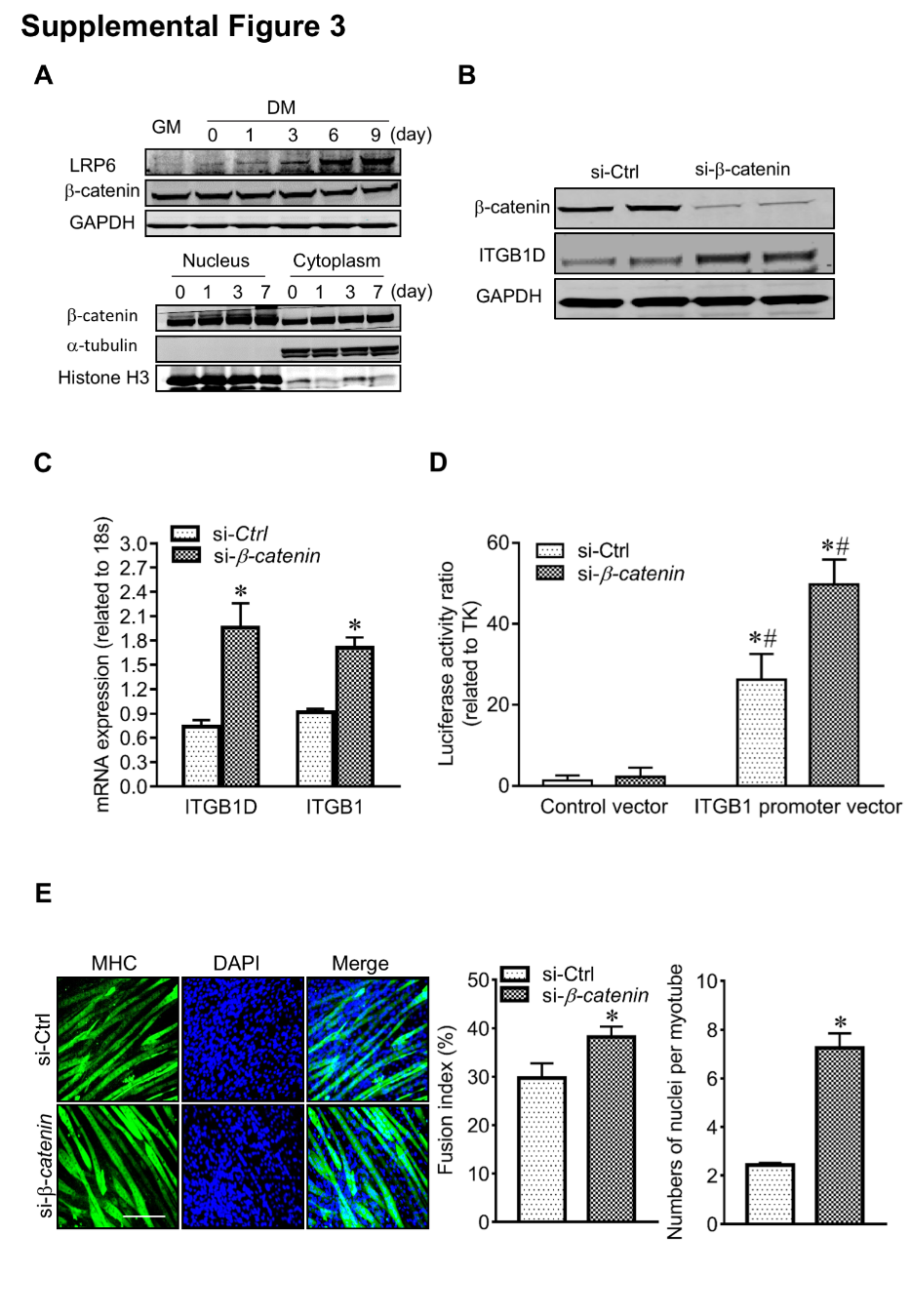


**Fig. S3.** β-catenin negatively regulates ITGB1D expression and myodifferentiation. (A) Western blotting examination of β-catenin protein during myodifferentiation of C2C12 myoblasts. GM: growth medium; DM: differentiation medium. The α-tubulin and histone H3 were used as the cytoplasmic and nuclear markers, respectively. (B) Effects of β-catenin on ITGB1D protein expression in neonatal myocytes. The proteins were examined after the treatment with β-catenin siRNAs for 48 hours. (C) qPCR analysis of ITGB1 and ITGB1D mRNAs expression of neonatal cardiomyocytes in the absence of β-catenin. *P < 0.05 compared with si-Ctrl. (D) Luciferase assay for the transcription of ITGB1. β-catenin-deficient HeLa cells were transfected with a ITGB1 promoter reporter construct for 24 h before activity measurement. *P < 0.05 compared with si-Ctrl; #P < 0.05 compared with groups other than si-Ctrl. (E) Effects of β-catenin on myodifferentiation of C2C12 myoblasts. β-catenin knockdown in C2C12 cells using β-catenin siRNAs did not affect cell viability but promoted the myotube fusion. Left, immunofluorescent microscopy of differentiating C2C12 myoblasts by day 6; right, measurement of myotube maturation. Scale bar: 10 μm. *P < 0.05 compared with si-Ctrl. The presented data and representative images from three to five independent experiments with similar results are shown.

**Supplementary Table 1.** Collection of primer sequences for RIP experiments, detection of ITGB1A and ITGB1D mRNA, silencing experiments, vector construction and RNA-seq validation.

**Supplementary Table 2.** Analysis of RNA-seq of cellular transcriptomes in human and rat.


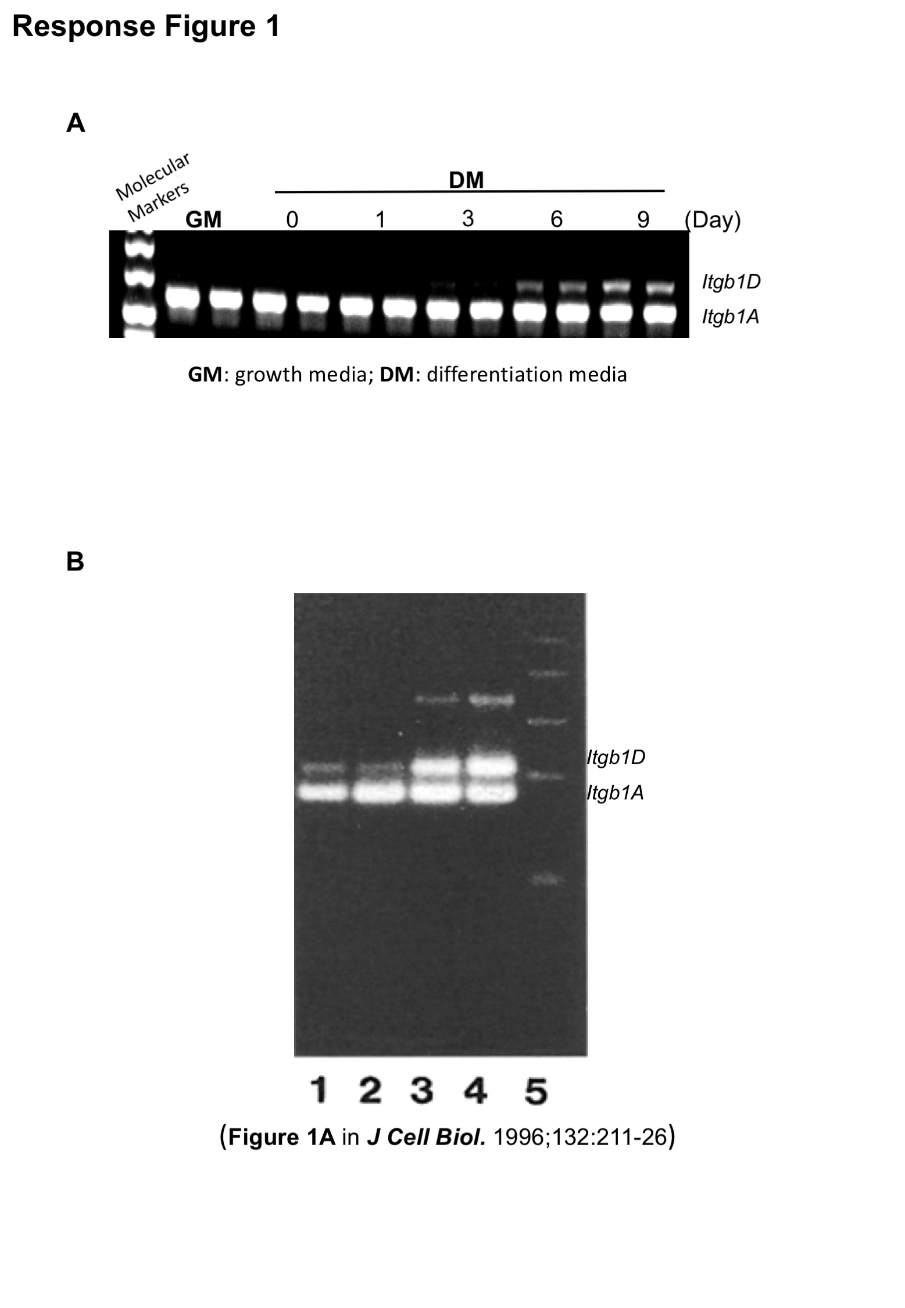


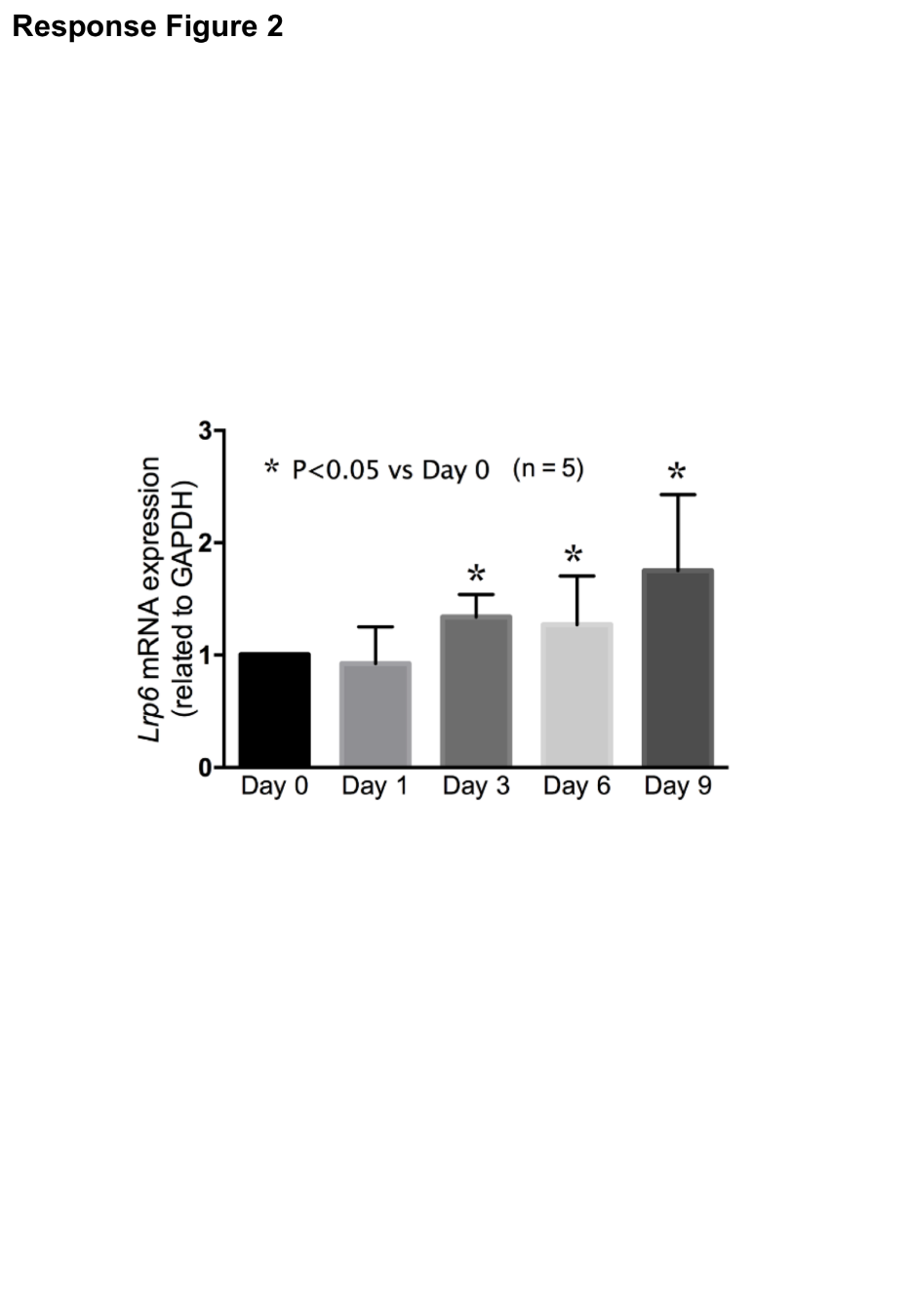

Supplement: Supplementary file 3 [file JCMM-22-4653-s003.docx]
